# Supplementary material for: Stepwise differentiation of follicular helper T cells reveals distinct developmental and functional states
Source: Nat Commun. 2023 Nov 24;14:7712. doi: 10.1038/s41467-023-43427-4 (PMC10674016; doi:10.1038/s41467-023-43427-4)
Supplement: Supplementary file 1 — Supplementary Information [file 41467_2023_43427_MOESM1_ESM.pdf]

Supplemental Figure 1

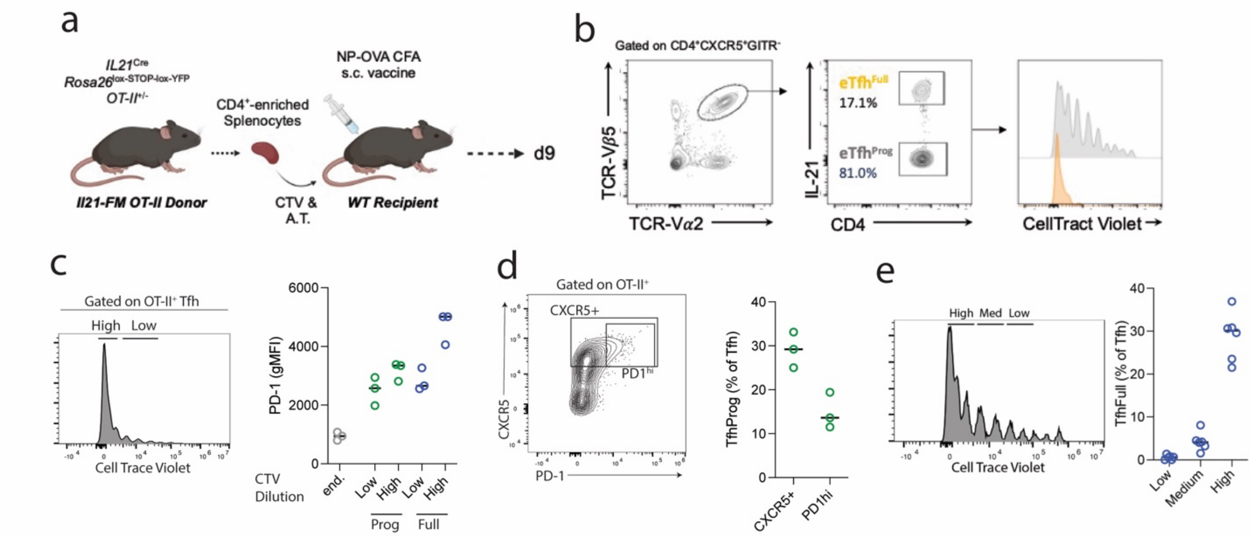

Figure S1. Proliferation and PD-1 expression of antigen-specific Tfh subsets. a) Experimental design and **b)** gating strategy to track cell division of antigen-specific Tfh subsets *in vivo*. **c)** Assessment of PD-1 expression on either Tfh-Prog or Tfh-Full gated on either high or low cell trace violet dilution (n=3 per group). **d)** Tfh-Prog frequency of total Tfh from either the total $OTII^+CXCR5^+CD4^+$ or $OTII^+CXCR5^+CD4^+PD1^{hi}$ gate (n=3 per group). **e)** Frequency of Tfh-Full of total Tfh cells in either high, medium, or low cell trace violet dilution gates (n=6 per group). Data are from a single experiment and are representative of n=2 independent experiments. Lines in plots indicate mean values.

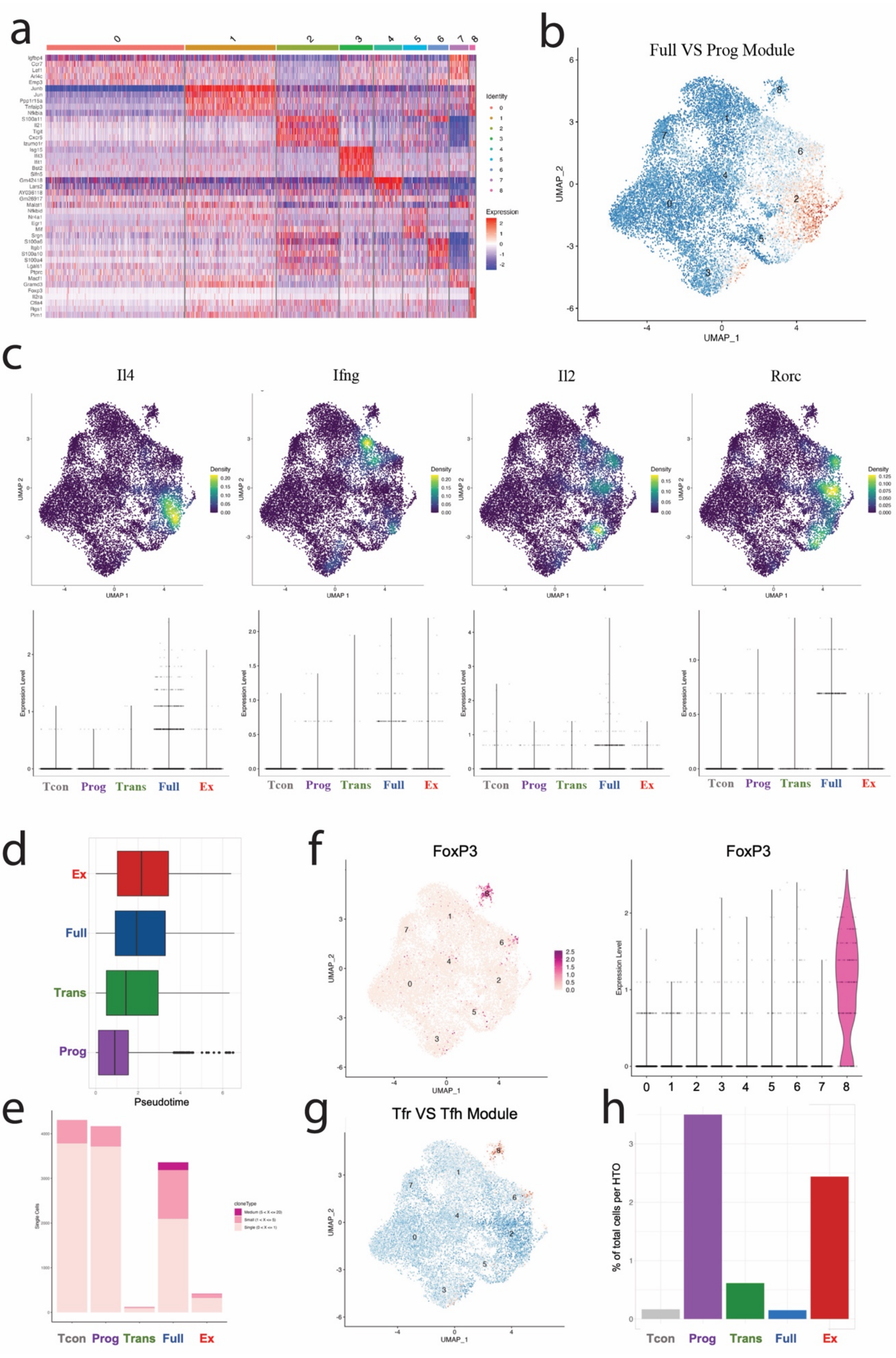
a
0
1
2
3
4
5
6
7
8
Igfbp4
Ccr7
Lef1
Arl4c
Emp3
Junb
Jun
Ppp1r15a
Tnfaip3
Nfkbia
S100a11
Il21
Tigit
Cxcr5
Izumo1r
Isg15
Ifit3
Ifit1
Bst2
Slfn5
Gm42418
Lars2
AY036118
Gm26917
Malat1
Nfkbid
Nr4a1
Egr1
Mif
Srgn
S100a6
Itgb1
S100a10
S100a4
Lgals1
Ptprc
Macf1
Gramd3
Foxp3
Il2ra
Ctla4
Rgs1
Prm1
Identity
Expression
b
Full VS Prog Module
UMAP_1
UMAP_2
c
Il4
Ifng
Il2
Rorc
Density
UMAP 1
UMAP 2
Expression Level
Tcon
Prog
Trans
Full
Ex
d
Ex
Full
Trans
Prog
Pseudotime
e
Single Cells
cloneType
Medium (5 < X <= 20)
Small (1 < X <= 5)
Single (0 < X <= 1)
Tcon
Prog
Trans
Full
Ex
f
FoxP3
UMAP_1
UMAP_2
FoxP3
Expression Level
g
Tfr VS Tfh Module
UMAP_1
UMAP_2
h
% of total cells per HTO
Tcon
Prog
Trans
Full
Ex

Figure S2. Transcriptional and clonal diversity of Tfh subsets. a) Top 5 upregulated genes in each UMAP cluster identified in the single-cell RNA seq dataset. **b)** Feature plot of the Full vs Prog module score applied on all cells post-filtering. **c)** Density and Violin plots showing the expression of selected cytokine-related transcripts. **d)** Boxplot representation of pseudotime scores in each hashtag. **e)** Clonotype distribution across hashtags. **f)** Feature and Violin plots showing Foxp3 expression at the single cell level within each cluster. **g)** Feature plot showing the module score for Tfr vs Tfh cells (derived from previous bulk RNA seq datasets). **h)** Percentage of Foxp3-expressing cells within each HTO.

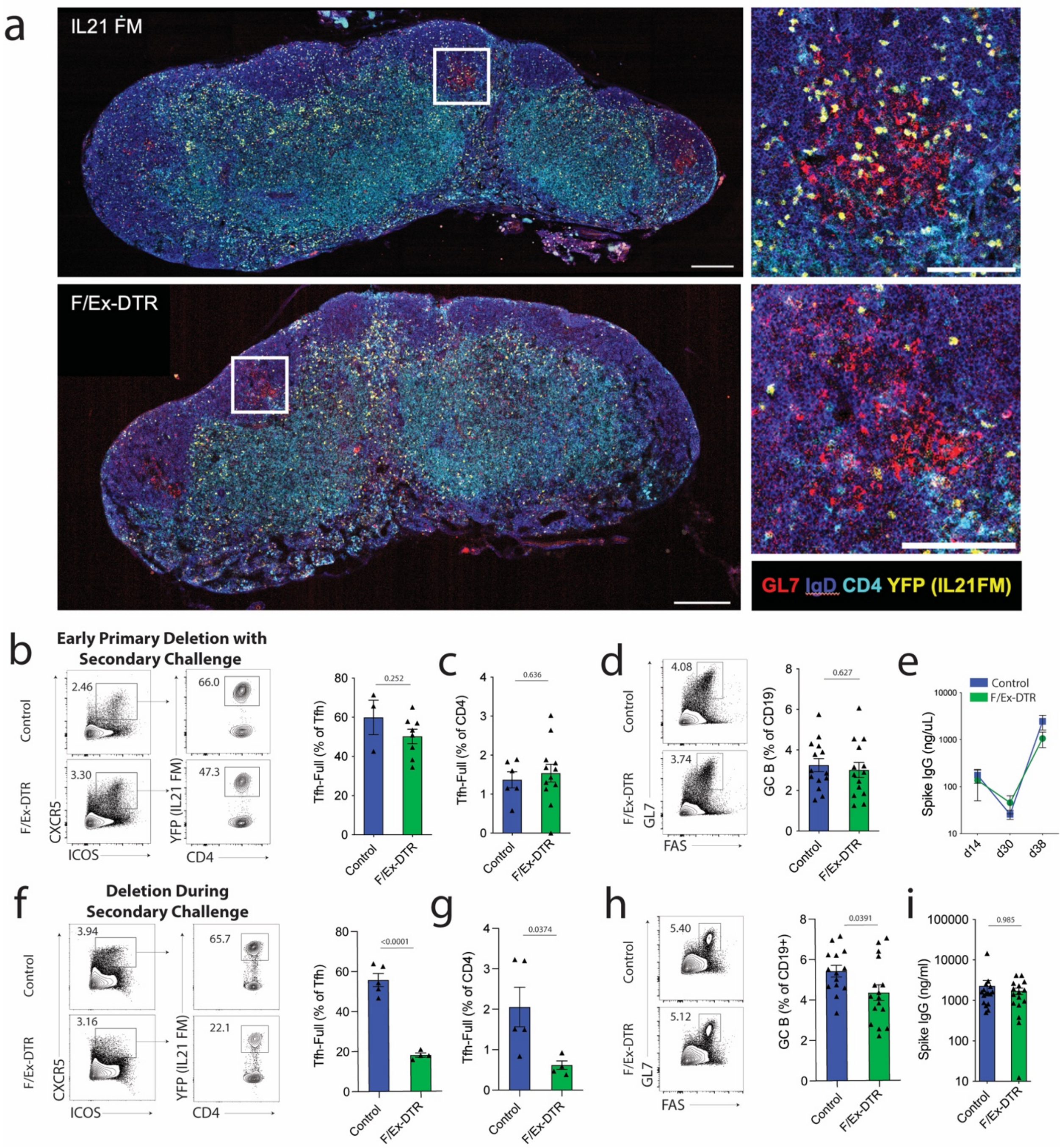

a
IL21 FM
F/Ex-DTR
GL7 IgD CD4 YFP (IL21FM)
b
Early Primary Deletion with Secondary Challenge
Control
F/Ex-DTR
2.46
3.30
66.0
47.3
CXCR5
ICOS
YFP (IL21 FM)
CD4
Tfh-Full (% of Tfh)
0.252
c
Tfh-Full (% of CD4)
0.636
d
4.08
3.74
GL7
FAS
GC B (% of CD19)
0.627
e
Control
F/Ex-DTR
Spike IgG (ng/uL)
d14
d30
d38
f
Deletion During Secondary Challenge
3.94
3.16
65.7
22.1
<0.0001
g
0.0374
h
5.40
5.12
GC B (% of CD19+)
0.0391
i
Spike IgG (ng/ml)
0.985

Figure S3. Phenotypic consequences of timed Tfh-F/Ex deletion. a) IL21FM and F/Ex-DTR ($Il21^{Cre}Rosa26^{L\text{-}S\text{-}L\text{-}YFP}Cxcr5^{L\text{-}S\text{-}L\text{-}DTR}$) mice were immunized subcutaneously with NP-OVA, given diphtheria toxin at 3, 6 and 8 days post-immunization, and draining LN were harvested after 9 days. Immunofluorescence staining shows efficient deletion of YFP^{+} Tfh cells from the B cell follicle (IgD^{+}) and the germinal center ($GL7^{+}$) areas. Representative micrographs of n=5 LNs analyzed. Scale bars = 250µM (left) and 100µM (right). **b)** Gating strategy (left) and frequency of Tfh-Full/Ex (right) cells after secondary challenge in mice with F/Ex deletion during primary immunization (see Fig 5k for experimental design) (n=3, Control; n=8, DTR). **c)** Frequency of Tfh-Full/Ex out of all $CD4^{+}$ T cells (n=6, Control; n=12, DTR), **d)** frequency of GC B cells (n=14, Control; n=14, DTR) and **e)** spike-specific serum IgG levels over time (n=7, Control; n=9, DTR). **f)** Gating strategy (left) and frequency of Tfh-Full/Ex (right) cells after deletion of F/Ex cells during secondary challenge (see Fig 5l for experimental design) (n=5, Control; n=4, DTR). **g)** Frequency of Tfh-Full/Ex out of all $CD4^{+}$ T cells (n=5, Control; n=4, DTR), **h)** frequency of GC B cells (n=14, Control; n=4, DTR) and **i)** spike-specific serum IgG levels at harvest (n=16, Control; n=16, DTR). Data are combined from n=2 independent experiments. P-values were calculated with unpaired two-tailed Student's *t*-tests. Data are represented as mean ± s.e.m in all plots.

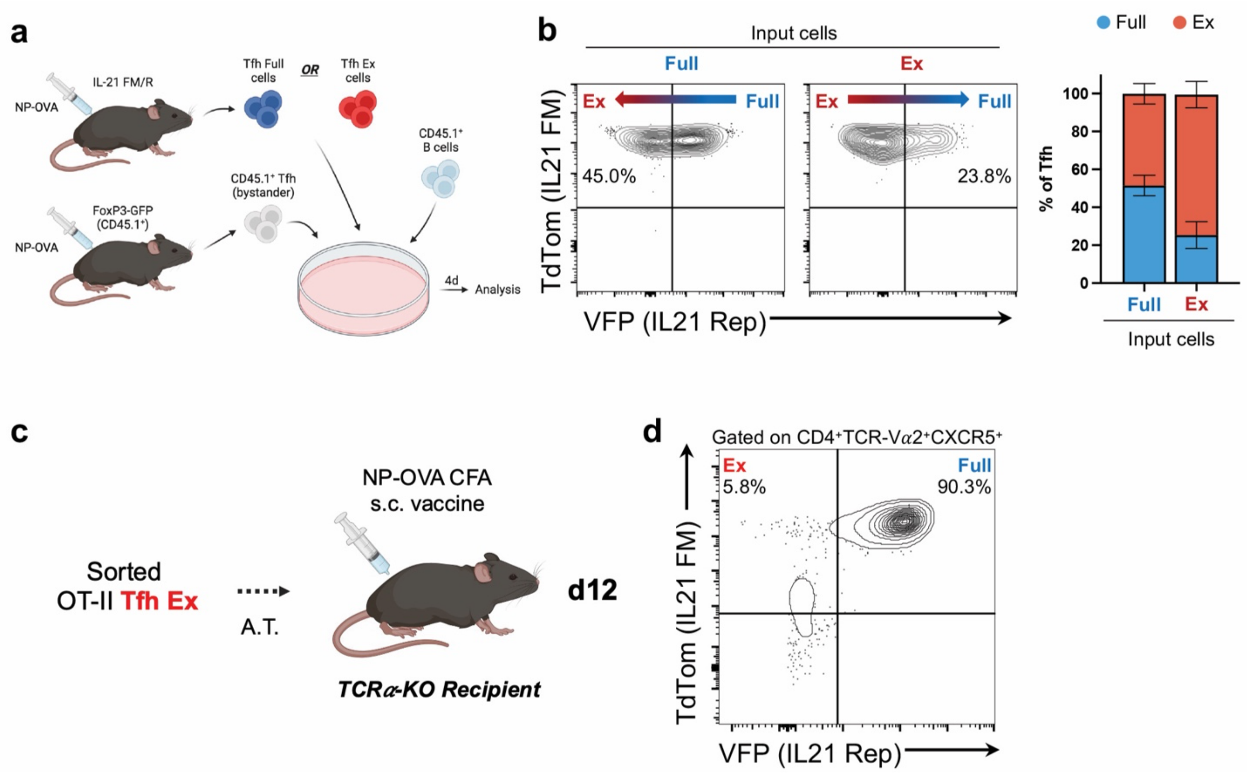

Figure S4. Plasticity of Tfh-Full/Ex subsets. a) Experimental setup for the in vitro analysis of IL21 plasticity in TfhFull/Ex subsets. IL21FM/R mice ($Il21^{Cre}Rosa26^{L\text{-}STOP\text{-}L\text{-}TdTomato}Il21^{VFP}$) were immunized with NP-OVA, and Tfh-Full ($CD4^{+}CD19^{-}GITR^{-}CXCR5^{+}TdTom^{+}VFP^{+}$) / Tfh-Ex ($CD4^{+}CD19^{-}GITR^{-}CXCR5^{+}TdTom^{+}VFP^{-}$) cells were sorted after 7 days; cells were cultured with B cells and bystander Tfh cells from a congenic mouse (CD45.1) and harvested 4 days post-culture. **b)** Representative gates and cumulative frequency of IL21 downregulation in Tfh-Full cells and IL21 upregulation in Tfh-Ex cells post-culture (n=6 per group). Data are represented as mean ± SD. **c)** $CD4^{+}$ splenocytes from an IL21FM/R OT-II mouse ($Il21^{Cre}Rosa26^{L\text{-}STOP\text{-}L\text{-}TdTomato}Il21^{VFP}OT\text{-}II^{+}$) were adoptively transferred into a WT recipient immunized with NP-OVA; Tfh-Ex OT-II cells ($TCR\text{-}V\alpha2^{+}TCR\text{-}V\beta5^{+}CXCR5^{+}TdTom^{+}VFP^{-}$) were sorted after 5 days and adoptively transferred into a $TCR\alpha^{-/-}$ recipient immunized with NP-OVA. **d)** Phenotype of adoptively transferred Tfh-Ex OT-II cells 12 days after immunization.

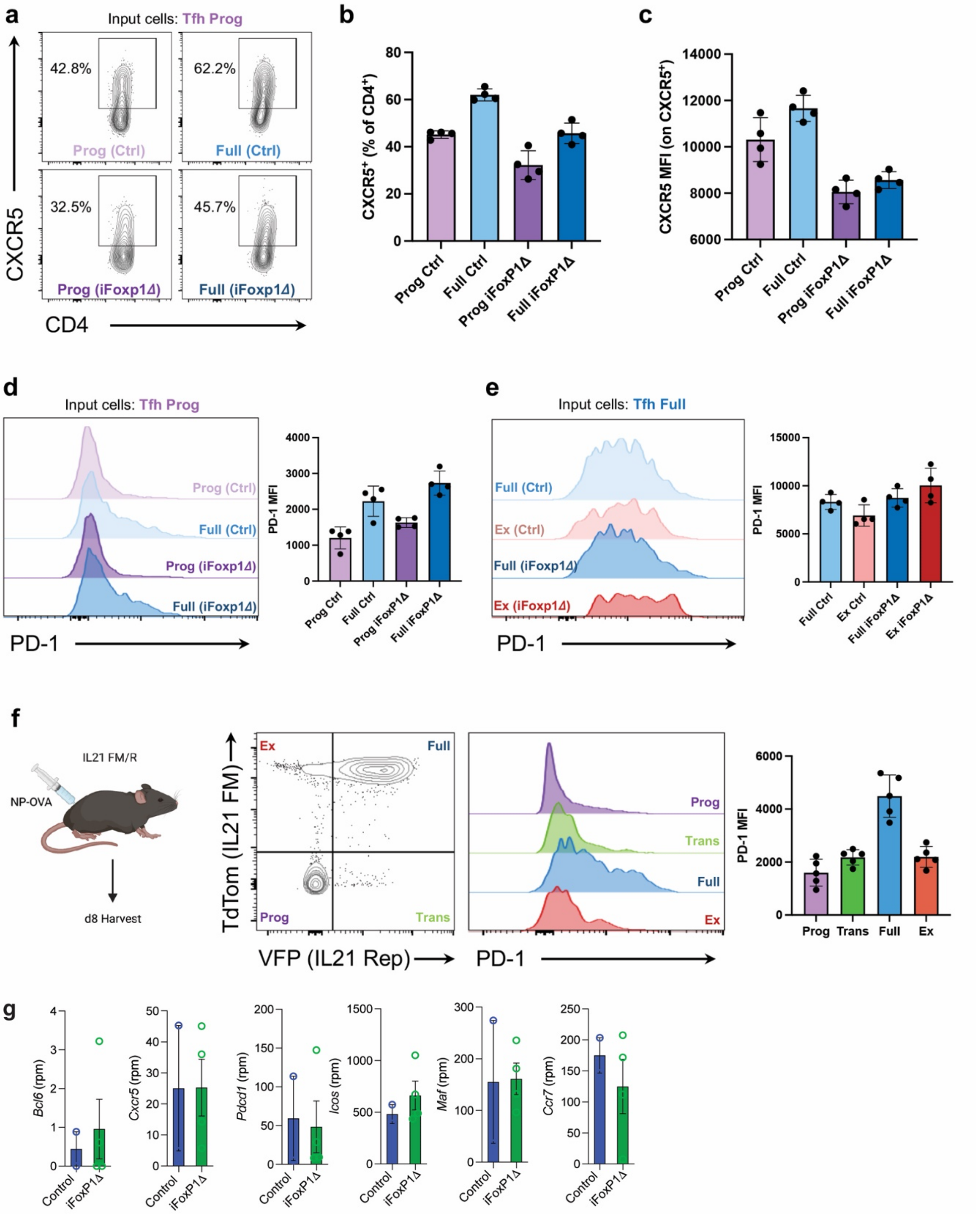

h

source	term_name	term_id	adjusted_p_value
GO:CC	intracellular membrane-bounded organelle	GO:0043231	5.68721E-06
GO:CC	membrane-bounded organelle	GO:0043227	2.88245E-05
GO:CC	intracellular organelle	GO:0043229	3.18599E-05
TF	Factor: E2F-1; motif: GNGGGCGGGRMN	TF:M10209	5.59362E-05
GO:CC	organelle	GO:0043226	9.22779E-05
GO:CC	intracellular anatomical structure	GO:0005622	0.000294381
TF	Factor: E2F-4; motif: GCGGGAAANA	TF:M02090	0.001070802
TF	Factor: E2F; motif: TTTSGCGSG	TF:M00918	0.002252993
GO:BP	organic substance biosynthetic process	GO:1901576	0.002525278
TF	Factor: FOXN4; motif: NNWANNCGWMCGCGTCNNNNMT	TF:M04662	0.002675962
TF	Factor: ZF5; motif: GSGCGCGR	TF:M00716	0.003041137
GO:BP	biosynthetic process	GO:0009058	0.003744723
GO:BP	cellular biosynthetic process	GO:0044249	0.005132088
TF	Factor: E2F; motif: GGCGSG; match class: 1	TF:M00803_1	0.005437277
TF	Factor: E2F; motif: NCSCGCSAAAN	TF:M00919	0.005642968

source	term_name	term_id	adjusted_p_value
TF	Factor: BEN; motif: CAGCGRNV	TF:M01240	0.006857936
TF	Factor: E2F-7; motif: GRGGCGGGAANNN	TF:M10212	0.007097847
TF	Factor: ZF5; motif: GSGCGCGR; match class: 1	TF:M00716_1	0.008263804
TF	Factor: E2F; motif: NKCGCGCSAAAN	TF:M00920	0.008629251
TF	Factor: IRF6; motif: NNNACTCYCGGKNNN	TF:M02874	0.01049159
TF	Factor: E2F; motif: GGCGSG	TF:M00803	0.01333638
TF	Factor: Kaiso; motif: SARNYCTCGCGAGAN	TF:M10276	0.013872565
TF	Factor: PEA3; motif: RCCGGAAGYN	TF:M01991	0.018535747
TF	Factor: E2F; motif: TTTSGCGSG	TF:M00939	0.019002673
TF	Factor: E2F-1; motif: TTTSGCGS	TF:M00431	0.025569023
TF	Factor: ELF4; motif: CCCGGAARTN	TF:M01979	0.025763804
GO:CC	nucleoplasm	GO:0005654	0.026853431
TF	Factor: FOXN4; motif: NNWANNCGWMCGCGTCNNNNMT;	TF:M04662_1	0.033379612
GO:CC	tertiary granule	GO:0070820	0.042459204
CORUM	Grin2b-Lrp8 complex	CORUM:5448	0.049957536

Figure S5. CXCR5 and PD-1 expression across Tfh subsets after FoxP1 deletion. a-c) Frequency of $CXCR5^{+}$ cells and median fluorescence intensity of CXCR5 in post-culture Tfh-Prog and de novo differentiated Tfh-Full from control or iFoxp1*Δ* mice (as detailed in Fig 7A) (n=4). **d)** PD-1 median fluorescence intensity in post-culture Tfh-Prog and de novo differentiated Tfh-Full from control and iFoxp1*Δ* mice (n=4). **e)** PD-1 median fluorescence intensity in post-culture Tfh-Full and de novo differentiated Tfh-Ex from control and iFoxp1*Δ* mice (see Figure 7A-C) (n=4). **f)** Ex-vivo analysis of PD-1 expression in IL21FM/R mice ($Il21^{Cre}Rosa26^{L\text{-}STOP\text{-}L\text{-}TdTomato}Il21^{VFP}$) 8 days after immunization with NP-OVA; PD-1 median fluorescence intensity was assessed on each Tfh subset separately (n=5). **g)** Gene expression levels in Tfh-Full (VFP+) from control and iFoxp1*Δ* mice through bulk RNASeq transcriptional analyses. **h)** Pathway analysis performed using differentially expressed genes (DEG) with higher expression on Foxp1 deleted Tfh-Full cells (Control; n=2, Deleter; n=4) utilizing gProfiler software. Data are represented as mean ± s.e.m in all plots.

a

source	term_name	term_id	adjusted_p_value
TF	Factor: ZF5; motif: GSGCGCGR	TF:M00716	1.11E-23
TF	Factor: ZF5; motif: GSGCGCGR; match class: 1	TF:M00716_1	7.41E-22
TF	Factor: E2F; motif: GGCGSG	TF:M00803	1.03E-21
TF	Factor: FOXN4; motif: NNWANNCGWMCGCGTCNNNNMT	TF:M04662	7.51E-21
TF	Factor: E2F-1; motif: GNGGGCGGGRMN	TF:M10209	2.08E-20
TF	Factor: FOXN4; motif: NNWANNCGWMCGCGTCNNNNMT; match class: 1	TF:M04662_1	9.97E-20
TF	Factor: ZF5; motif: NRNGNGCGCGCWN; match class: 1	TF:M00333_1	1.01E-18
TF	Factor: ELK-1; motif: ACCGGAWRTN; match class: 1	TF:M01981_1	4.05E-18
TF	Factor: PEA3; motif: RCCGGAAGYN; match class: 1	TF:M01991_1	8.84E-18
TF	Factor: Kaiso; motif: SARNYCTCGCGAGAN	TF:M10276	1.04E-17
TF	Factor: GABP-alpha; motif: CTTCCK	TF:M01660	1.13E-17
TF	Factor: ZF5; motif: NRNGNGCGCGCWN	TF:M00333	4.99E-17
TF	Factor: ZF5; motif: GYCGCGCARNGCNN	TF:M02933	2.40E-15
TF	Factor: E2F-1; motif: GNGGGCGGGRMN; match class: 1	TF:M10209_1	4.10E-15
TF	Factor: ELK-1; motif: ACCGGAWRTN	TF:M01981	6.79E-15
TF	Factor: Elk-1; motif: NNNNCCGGAARTNN	TF:M00025	4.54E-14
TF	Factor: PEA3; motif: RCCGGAAGYN	TF:M01991	1.72E-13
TF	Factor: c-ets-1; motif: ACCGGAWRYN	TF:M01986	2.51E-13
TF	Factor: Foxn1; motif: RGAMGC	TF:M12470	4.29E-13
TF	Factor: E2F-3; motif: GGCGGGN	TF:M02089	1.02E-12
TF	Factor: Elk-1; motif: NNNNCCGGAARTNN; match class: 1	TF:M00025_1	2.51E-12

source	term_name	term_id	adjusted_p_value
TF	Factor: Elk-1; motif: NCCGGAAGTGN	TF:M10219	4.38E-12
TF	Factor: TEL1; motif: CNCGGAANNN	TF:M01993	5.45E-12
TF	Factor: XBP-1; motif: WNNGMCACGTC	TF:M01770	5.68E-12
TF	Factor: Kaiso; motif: SARNYCTCGCGAGAN; match class: 1	TF:M10276_1	1.06E-11
TF	Factor: ERG; motif: ACCGGAART	TF:M01752	2.24E-11
TF	Factor: Elk-1; motif: NCCGGAAGTGN; match class: 1	TF:M10219_1	2.24E-11
TF	Factor: E2F-4; motif: NTTTCSCGCC	TF:M07380	2.45E-11
TF	Factor: ERG; motif: ACCGGAART; match class: 1	TF:M01752_1	3.22E-11
TF	Factor: c-Ets-1; motif: NNNRCCGGAWRYNNNN	TF:M01078	9.17E-11
TF	Factor: BEN; motif: CAGCGRNV; match class: 1	TF:M01240_1	1.03E-10
TF	Factor: BCL6B; motif: NNNNCCGCCCCWNNNN	TF:M02844	1.22E-10
TF	Factor: Ets2; motif: ACCGGAWRYN	TF:M01989	1.31E-10
TF	Factor: SAP-1a; motif: NRRCCGGAAGYRN	TF:M10220	2.14E-10
TF	Factor: Ets2; motif: ACCGGAWRYN; match class: 1	TF:M01989_1	2.42E-10
TF	Factor: E2F-4; motif: GCGGGAAANA	TF:M02090	6.34E-10
TF	Factor: Sp1; motif: NGGGGCGGGGN	TF:M07395	6.55E-10
TF	Factor: GABP-alpha; motif: CTTCCK; match class: 1	TF:M01660_1	8.52E-10
TF	Factor: BEN; motif: CAGCGRNV	TF:M01240	8.85E-10
TF	Factor: ELF4; motif: CCCGGAARTN	TF:M01979	1.15E-09
TF	Factor: E2F-1; motif: NKTSSCGC	TF:M00428	1.21E-09

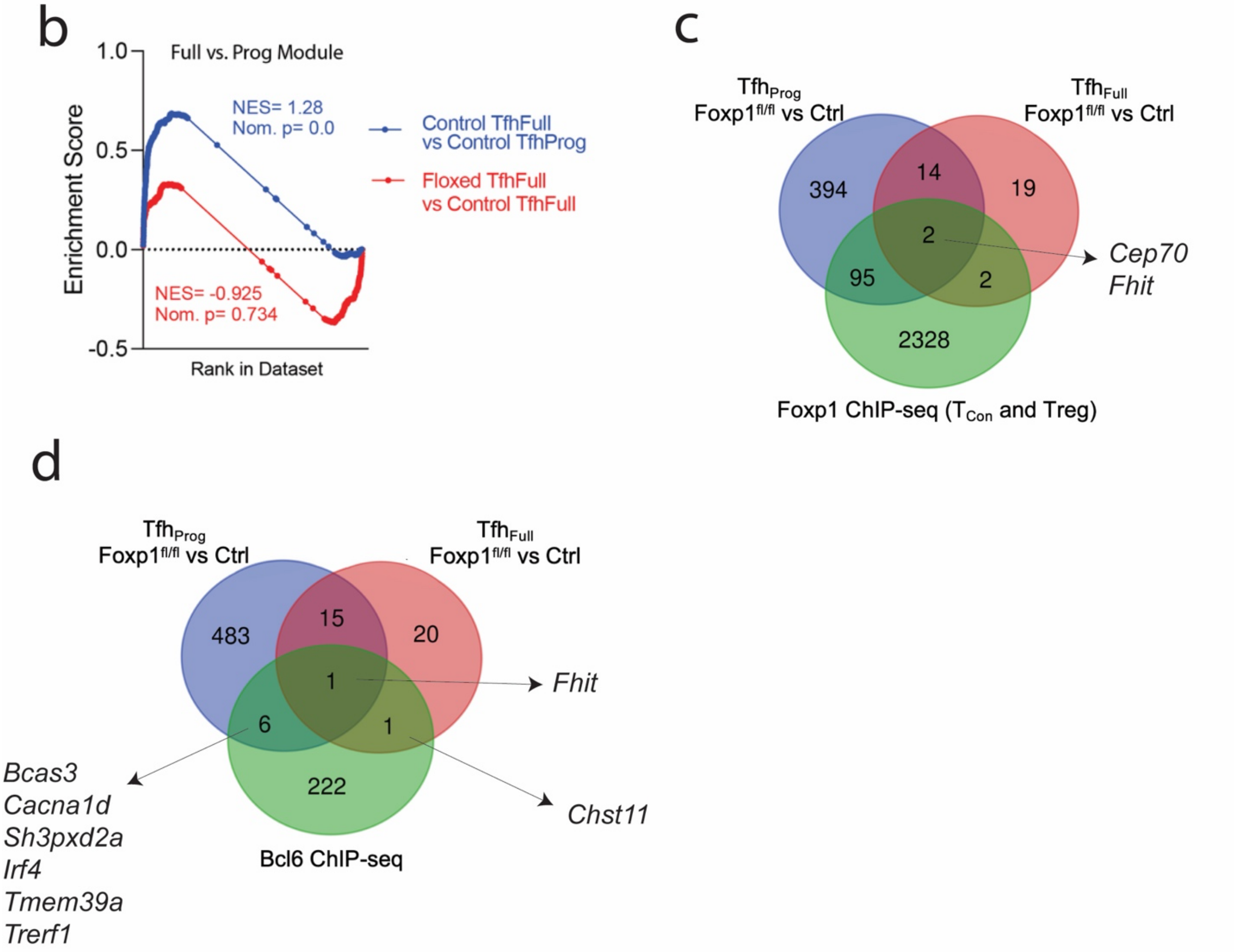

Figure S6. Transcriptomic changes in response to FoxP1 deletion in Tfh subsets. a) Analysis of transcription factor binding motif enrichment from differentially expressed genes between control or FoxP1-deleted Tfh-Prog cells. **b)** GSEA of a Tfh-Full gene module (as in Fig. 3f) in Tfh-Full from iFoxp1Δ and control mice. NES= normalized enrichment score. *P* value was calculated using empirical phenotype-based permutation tests. **c)** Venn diagram showing the overlap between DEGs from iFoxp1Δ and control Tfh-Full/Tfh-Prog (adjusted $p<0.05$) and a previously published FoxP1 ChIP-seq dataset[33]. **d)** Venn diagram showing the overlap between DEG from iFoxp1Δ and control Tfh-Full/Tfh-Prog and a previously published Bcl6 ChIP-seq dataset[35].
