# Supplementary material for: Stepwise differentiation of follicular helper T cells reveals distinct developmental and functional states
Source: Nat Commun. 2023 Nov 24;14:7712. doi: 10.1038/s41467-023-43427-4 (PMC10674016; doi:10.1038/s41467-023-43427-4)
Supplement: Supplementary file 3 — Reporting Summary [file 41467_2023_43427_MOESM3_ESM.pdf]

nature portfolio

Corresponding author(s): Peter Sage

Last updated by author(s): Oct 19, 2023

Reporting Summary

Nature Portfolio wishes to improve the reproducibility of the work that we publish. This form provides structure for consistency and transparency in reporting. For further information on Nature Portfolio policies, see our Editorial Policies and the Editorial Policy Checklist.

Statistics

For all statistical analyses, confirm that the following items are present in the figure legend, table legend, main text, or Methods section.

n/a	Confirmed	
☐	☒	The exact sample size (*n*) for each experimental group/condition, given as a discrete number and unit of measurement
☒	☐	A statement on whether measurements were taken from distinct samples or whether the same sample was measured repeatedly
☐	☒	The statistical test(s) used AND whether they are one- or two-sided<br>*Only common tests should be described solely by name; describe more complex techniques in the Methods section.*
☒	☐	A description of all covariates tested
☐	☒	A description of any assumptions or corrections, such as tests of normality and adjustment for multiple comparisons
☐	☒	A full description of the statistical parameters including central tendency (e.g. means) or other basic estimates (e.g. regression coefficient) AND variation (e.g. standard deviation) or associated estimates of uncertainty (e.g. confidence intervals)
☐	☒	For null hypothesis testing, the test statistic (e.g. *F*, *t*, *r*) with confidence intervals, effect sizes, degrees of freedom and *P* value noted<br>*Give P values as exact values whenever suitable.*
☒	☐	For Bayesian analysis, information on the choice of priors and Markov chain Monte Carlo settings
☒	☐	For hierarchical and complex designs, identification of the appropriate level for tests and full reporting of outcomes
☐	☒	Estimates of effect sizes (e.g. Cohen's *d*, Pearson's *r*), indicating how they were calculated

Our web collection on statistics for biologists contains articles on many of the points above.

Software and code

Policy information about availability of computer code

Data collection: No software was used for data collection.

Data analysis: Single-Cell RNA Sequencing: CellRanger was used for initial reads processing, and output data were loaded into R environment; additional R-based software packages were used for downstream analyses, including Seurat, SingleR, scRepertoire and Monocle3. RNA velocity analysis was carried out with scVelo and velocyto (Python).

Bulk RNA Sequencing: Analysis was performed using the CLC Genomics Workbench version 8.0.1 RNA-seq analysis software package (Qiagen) and DESeq2. Briefly, reads were aligned (mismatch cost=2, insertion cost=3, deletion cost=3, length fraction=0.8, similarity fraction=0.8) to the mouse genome. Gene counts were loaded in R environment and differential gene expression was assessed with DESeq2. Gene-e (Broad Institute) was used to generate heatmaps. G:Profiler was used for pathway analysis using default settings.

ATAC Sequencing:qquality trimming and primer removal from the raw fastq files were performed using Trimmomatic (v3.9) using the following parameters: LEADING:15 TRAILING:15 SLIDINGWINDOW:4:15 and MINLEN:36. The trimmed reads were aligned to Mm10 genome using Bowtie2 (v2.4.5) using a maximum insert size of 1,000. PCR duplicates were marked using Picard (2.18.7). The concordance of each biological condition was assessed by the average Pearson correlation across all pairwise combinations. Peak-calling was performed for each biological condition using MACS (v2.2.7.1) on merged bam files with a q-value threshold of 0.001. Consensus peaks from all biological conditions were then merged to create a single peak universe of 56,060 regions. Cut sites were extracted from each biological replicate and the number of cuts within each peak region was quantified (BEDtools v2.30.0) to generate a raw counts matrix. DESeq2 (v3.15) was used to normalize the counts matrix and perform differential accessibility analysis between all of the relevant comparisons. For any given comparison, an FDR cutoff of 0.05 was used to determine the differential ChARs. Gene-to-peak associations were determined using the GREAT software package (v3.0.0). ATAC-seq tracks were visualized using Integrative Genomics Viewer (v2.13.2). Motif enrichment analysis was performed

using HOMER (v3.0) with default settings.

For manuscripts utilizing custom algorithms or software that are central to the research but not yet described in published literature, software must be made available to editors and reviewers. We strongly encourage code deposition in a community repository (e.g. GitHub). See the Nature Portfolio guidelines for submitting code & software for further information.

Data

Policy information about availability of data

All manuscripts must include a data availability statement. This statement should provide the following information, where applicable:

- Accession codes, unique identifiers, or web links for publicly available datasets
- A description of any restrictions on data availability
- For clinical datasets or third party data, please ensure that the statement adheres to our policy

The datasets generated during the current study are available on GEO (GSE225724) and are available from the corresponding author on reasonable request.

Research involving human participants, their data, or biological material

Policy information about studies with human participants or human data. See also policy information about sex, gender (identity/presentation), and sexual orientation and race, ethnicity and racism.

Reporting on sex and gender	This study did not involve human participants.
Reporting on race, ethnicity, or other socially relevant groupings	This study did not involve human participants.
Population characteristics	This study did not involve human participants.
Recruitment	This study did not involve human participants.
Ethics oversight	This study did not involve human participants.

Note that full information on the approval of the study protocol must also be provided in the manuscript.

Field-specific reporting

Please select the one below that is the best fit for your research. If you are not sure, read the appropriate sections before making your selection.

[x] Life sciences [ ] Behavioural & social sciences [ ] Ecological, evolutionary & environmental sciences

For a reference copy of the document with all sections, see nature.com/documents/nr-reporting-summary-flat.pdf

Life sciences study design

All studies must disclose on these points even when the disclosure is negative.

Sample size	All experiments were performed at least twice. Sample size calculations were not formally carried out since effect sizes for most experiments were unknown a priori. Sample sizes were determined by genotype availability according to mendelian genetics and accounting for the age of mice.
Data exclusions	No data were excluded from the analyses.
Replication	All experimental findings were confirmed with at least two biological replicates.
Randomization	Age-matched or litter mates were included in each experimental group. Randomization was not performed because group designation was determined based on genetics.
Blinding	Blinding was not relevant to the study because groups were determined during downstream analysis based on genotype.

Reporting for specific materials, systems and methods

We require information from authors about some types of materials, experimental systems and methods used in many studies. Here, indicate whether each material, system or method listed is relevant to your study. If you are not sure if a list item applies to your research, read the appropriate section before selecting a response.

Materials & experimental systems

n/a	Involved in the study
	x Antibodies
	x Eukaryotic cell lines
x	Palaeontology and archaeology
	x Animals and other organisms
x	Clinical data
x	Dual use research of concern
x	Plants

Methods

n/a	Involved in the study
x	ChIP-seq
	x Flow cytometry
x	MRI-based neuroimaging

Antibodies

Antibodies used

Antibodies

anti-CD4 (Biolegend, clone RM4-5 or BD, clone GK1.5, both 1:200),
https://www.biolegend.com/en-us/products/percp-cyanine5-5-anti-mouse-cd4-antibody-4230
https://www.bdbiosciences.com/en-us/products/reagents/flow-cytometry-reagents/research-reagents/single-color-antibodies-ruo/buv395-rat-anti-mouse-cd4.563790

anti-CD19 (Biolegend, clone 6D5 or BD, clone 1D3, both 1:200),
https://www.biolegend.com/en-us/products/apc-cyanine7-anti-mouse-cd19-antibody-3903
https://www.bdbiosciences.com/en-us/products/reagents/flow-cytometry-reagents/research-reagents/single-color-antibodies-ruo/buv737-rat-anti-mouse-cd19.612782

anti-ICOS (Biolegend, 1:200, clone 15F9),
https://www.biolegend.com/en-us/products/pe-anti-mouse-cd278-icos-antibody-37?GroupID=BLG6978

anti-PD-1 (Biolegend, 1:200, clone RMP1-30),
https://www.biolegend.com/en-us/products/pe-cyanine7-anti-mouse-cd279-pd-1-antibody-3612

anti-CXCR5 (Biolegend, 1:200, clone L138D7),
https://www.biolegend.com/en-us/products/biotin-anti-mouse-cd185-cxcr5-antibody-8552

anti-GITR (Biolegend, 1:200, clone DTA-1),
https://www.biolegend.com/en-us/products/apc-anti-mouse-cd357-gitr-antibody-4646

anti-CD45.1 (Biolegend, 1:200, clone A20),
https://www.biolegend.com/en-us/products/alexa-fluor-700-anti-mouse-cd45-1-antibody-3392

anti-Fas (BD, 1:200, clone Jo2),
https://www.bdbiosciences.com/en-us/products/reagents/flow-cytometry-reagents/research-reagents/single-color-antibodies-ruo/pe-cy-7-hamster-anti-mouse-cd95.557653

anti-CD38 (Biolegend, 1:200, clone 90),
https://www.biolegend.com/en-us/products/pacific-blue-anti-mouse-cd38-antibody-6652?Clone=90

anti-IA/IE (BD, 1:1000, clone 2G9),
https://www.bdbiosciences.com/en-us/products/reagents/flow-cytometry-reagents/research-reagents/single-color-antibodies-ruo/buv395-rat-anti-mouse-i-a-i-e.569244

anti-FoxP3 (eBiosciences, 1:200, clone FJK-16s),
https://www.thermofisher.com/antibody/product/FOXP3-Antibody-clone-FJK-16s-Monoclonal/53-5773-82

anti-B220 (Biolegend, 1:200, clone RA3-6B2),
https://www.biolegend.com/en-us/products/pe-anti-mouse-human-cd45r-b220-antibody-447

T- and B-cell activation antigen (BD Biosciences, 1:200 for flow cytometry, 1:100 for microscopy, clone GL-7),
https://www.bdbiosciences.com/en-us/products/reagents/flow-cytometry-reagents/research-reagents/single-color-antibodies-ruo/fitc-rat-anti-mouse-t-and-b-cell-activation-antigen.553666

anti-CD138 (Biolegend, 1:200, clone 281-2),
https://www.biolegend.com/en-us/products/apc-anti-mouse-cd138-syndecan-1-antibody-7572?GroupID=BLG9623

Anti-TCR Va2 (Biolegend, 1:200, clone, B20.1),
https://www.biolegend.com/en-us/products/percp-cyanine5-5-anti-mouse-tcr-valpha2-antibody-4872

anti-TCR Vβ5.1, 5.2 (Biolegend, 1:200, clone MR9-4), https://www.biolegend.com/en-us/products/apcfire-750-anti-mouse-tcr-vbeta51-52-antibody-16237

anti-CD55 (Biolegend, 1:200, clone RIKO-3), https://www.biolegend.com/en-us/products/pe-anti-mouse-cd55-daf-antibody-5514?GroupID=BLG10436

anti-CD124 (BD Pharmingen, 1:200, mIL4R-M1), https://www.bdbiosciences.com/en-us/products/reagents/flow-cytometry-reagents/research-reagents/single-color-antibodies-ruo/pe-rat-anti-mouse-cd124.561695

anti-EBI2 (Biolegend, 1:200, clone SA313E4), https://www.biolegend.com/en-us/products/pe-anti-human-gpr183-ebi2-antibody-13931

anti-Ly6a (Biolegend, 1:200, clone D7), https://www.biolegend.com/en-us/products/pe-anti-mouse-ly-6a-e-sca-1-antibody-228?GroupID=BLG2524

anti-CD4 (rabbit anti-mouse, Abcam, 1:100, clone EPR19514), https://www.abcam.com/products/primary-antibodies/cd4-antibody-epr19514-ab183685.html

IgD (goat anti-mouse, 1:500 Novus Biologicals, polyclonal), https://www.novusbio.com/products/igd-fc-antibody_nbp2-69334

Secondary antibodies: minimally cross-reactive donkey anti-goat AlexaFluor-594 and donkey anti-rabbit AlexaFluor-647, both 1:1000, Jackson Immunoresearch),
GFP-Booster AlexaFluor-488 (1:500, ChromoTek),
alkaline phosphatase-conjugated anti-IgG secondary antibody (Southern Biotech, 1:1000).

Validation: All antibodies were utilized according to manufacturer validated applications and/or previously published applications.

Eukaryotic cell lines

Policy information about cell lines and Sex and Gender in Research

Cell line source(s): NB21.2D9 cells were kindly provided by Dr. Garnett Kelsoe.

Authentication: Cells were not authenticated.

Mycoplasma contamination: Cells were not tested for mycoplasma contamination.

Commonly misidentified lines (See ICLAC register): No commonly misidentified cell lines were used in the study.

Animals and other research organisms

Policy information about studies involving animals; ARRIVE guidelines recommended for reporting animal research, and Sex and Gender in Research

Laboratory animals: Mice from Jackson Laboratories (all on the C57BL/6 background): Rosa26Lox-STOP-Lox-YFP, Rosa26Lox-STOP-Lox-TdTomato, Il21VFP, Foxp3IRES-Cre-YFP, Ptprca, UBCCre-ERT2, OT-II, Foxp1fl/fl and Bcl6fl/fl.
Tg(Il21Cre) mice were a kind gift from Uta Hoepken and have been published previously (Wichner, K. et al. FASEB J 30, 761-774 (2016)).
Foxp3IRES-GFP mice and Cxcr5IRES-LoxP-STOP-LoxP-DTR mice have been published previously (Clement, R.L. et al. Nature immunology 20, 1360-1371 (2019) and Bettelli, E. et al. Nature 441, 235-238 (2006)).
All mice were 6-10 weeks old. Mice were kept at a 12/12h dark/light cycle, 22C ambient temperature, and 42% humidity.

Wild animals: This study did not involve wild animals.

Reporting on sex: Sex based-analysis were not performed. All experiments were performed with both males and females.

Field-collected samples: This study did not involve field-collected samples.

Ethics oversight: All animals were used according to Brigham and Women’s Hospital Institutional Animal Care and Use Committee policies, as well as the National Institute of Health guidelines.

Note that full information on the approval of the study protocol must also be provided in the manuscript.

Flow Cytometry

Plots

Confirm that:

- [x] The axis labels state the marker and fluorochrome used (e.g. CD4-FITC).
- [x] The axis scales are clearly visible. Include numbers along axes only for bottom left plot of group (a 'group' is an analysis of identical markers).
- [x] All plots are contour plots with outliers or pseudocolor plots.
- [x] A numerical value for number of cells or percentage (with statistics) is provided.

Methodology

Sample preparation	Draining lymph nodes or spleens were mashed through 70-micron filters and resuspended in PBS supplemented with 1% FBS and 1mM EDTA.
Instrument	Cytek AURORA (5-laser configuration), BD FACS Aria II cell sorter, BD FACS Symphony S6 sorter and CytoFLEX SRT sorter.
Software	All flow cytometry data was analyzed with FlowJo version 10.
Cell population abundance	Sort purity was checked whenever possible and was above 95% by flow cytometry.
Gating strategy	Gating strategies are described and shown throughout the manuscript.

- [x] Tick this box to confirm that a figure exemplifying the gating strategy is provided in the Supplementary Information.
